# Towards survival prediction of cancer patients using medical images

**DOI:** 10.7717/peerj-cs.1090

**Published:** 2022-10-26

**Authors:** Nazeef Ul Haq, Bilal Tahir, Samar Firdous, Muhammad Amir Mehmood

**Affiliations:** 1 Al-Khawarizmi Institute of Computer Science (KICS), University of Engineering and Technology (UET), Lahore, Pakistan; 2 King Edward Medical University (KEMU), Lahore, Pakistan

**Keywords:** Brain tumor, Medical imaging

## Abstract

Survival prediction of a patient is a critical task in clinical medicine for physicians and patients to make an informed decision. Several survival and risk scoring methods have been developed to estimate the survival score of patients using clinical information. For instance, the Global Registry of Acute Coronary Events (GRACE) and Thrombolysis in Myocardial Infarction (TIMI) risk scores are developed for the survival prediction of heart patients. Recently, state-of-the-art medical imaging and analysis techniques have paved the way for survival prediction of cancer patients by understanding key features extracted from Magnetic Resonance Imaging (MRI) and Computed Tomography (CT) scanned images with the help of image processing and machine learning techniques. However, survival prediction is a challenging task due to the complexity in benchmarking of image features, feature selection methods, and machine learning models. In this article, we evaluate the performance of 156 visual features from radiomic and hand-crafted feature classes, six feature selection methods, and 10 machine learning models to benchmark their performance. In addition, MRI scanned Brain Tumor Segmentation (BraTS) and CT scanned non-small cell lung cancer (NSCLC) datasets are used to train classification and regression models. Our results highlight that logistic regression outperforms for the classification with 66 and 54% accuracy for BraTS and NSCLC datasets, respectively. Moreover, our analysis of best-performing features shows that age is a common and significant feature for survival prediction. Also, gray level and shape-based features play a vital role in regression. We believe that the study can be helpful for oncologists, radiologists, and medical imaging researchers to understand and automate the procedure of decision-making and prognosis of cancer patients.

## Introduction

Medical imaging has been a highly adopted technique for diagnostics in the healthcare sector due to its speed, accuracy, and non-invasiveness. According to the National Health Service (NHS), in 2020, 40.4 million medical imaging tests are performed in United Kingdom (England & Improvement, 2016). Similarly, the global market size of medical imaging is estimated to be $26 billion in 2021 with expected growth to $35 billion by 2026 (Markets, 2021). This adaption of different medical imaging modalities like positron emission tomography (PET) (Bailey et al., 2005), magnetic resonance imaging (MRI) (Lam, 2018), and computed tomography (CT) scan (De Chiffre et al., 2014) by doctors is attributed to their aid in better diagnosis, treatment, and prognosis. In general, these medical images are used to diagnose heart disease, lung disease, Coronavirus disease 2019 (COVID-19), fractured bones, cancer, and brain disorders (Sharma & Aggarwal, 2010). Moreover, Artificial Intelligence (AI) based computer-aided diagnosis (CADx) algorithms are providing additional advantage of automatically identifying and interpreting the anomalies from images to assist doctors in clinical diagnostics and prognosis. One key aspect of such clinical prognosis is survival prediction of patients using information like age, sex, disease history, and profile. In this regard, researchers have developed different scoring techniques like thrombolysis in myocardial infarction (TIMI) risk (Antman et al., 2000), Global Registry of Acute Coronary Events (GRACE) (Tang, Wong & Herbison, 2007), NSTEMI (non-ST-segment elevation myocardial infarction), Pitt bacteremia Score (PBS) (Henderson et al., 2020; Marchesini, Morelli & Piangerelli, 2015), and acute physiology and chronic health evaluation II (APACHE II) (Park et al., 2009) to predict the survival time of patients. These scoring methods are designed after experimenting and selecting the most important factors contributing to the survival of the patient (Widera et al., 2012). In addition to clinical information, medical images also provide a novel opportunity to predict the survival rate of cancer patients after examining the complex visual features of tumorous cells. In general, different types of radiomic and hand-crafted features of the image are extracted to examine the relationship between visual features and survival rate of patient (Banerjee, Mitra & Shankar, 2018; Alam et al., 2018). For instance, *shape-based* and *first-order* based radiomic features are used for survival prediction of brain tumor patients form BraTS dataset (Sun & Zhang, 2018; Banerjee, Mitra & Shankar, 2018). Similarly, *hand-crafted* features of tumor like volume, volume ratio, and position, etc., of tumor cells are introduced for survival prediction (Guo et al., 2019; Banerjee, Mitra & Shankar, 2018). However, these studies are limited to specific pre-selected feature types and machine learning models. It’s inevitable that someone will do a major review of different features, feature selection methods, and machine learning models.

In this article, we test the performance of 10 machine learning models with gold standard BraTS and NSCLC datasets containing brain tumor and lung cancer images, respectively. Also, we extract and compare the performance of nine classes of radiomic and hand-crafted features of medical images. Precisely, we evaluate a total of 540 combinations formulated by using eight feature classes, six feature selection methods, and 10 machine learning models. For classification, we find that Logistic Regression (LoR) outperforms competing models on MRI and CT scanned images. LoR model gives the maximum 66% and 54% accuracy on BraTS and NSCLC datasets, respectively. Similarly, the Linear Regression (LR) model is the best performing model for regression with 52–53% accuracy on both datasets. Moreover, age is the most important factor in the survival prediction of cancer patients. Our major contributions and findings are as follows:

• We evaluate the performance of 156 image features from nine classes, six feature selection methods, and 10 machine learning models to select the best performing model and features for MRI and CT scanned datasets of BraTS and NSCLC, respectively.
• A comprehensive analysis using the AUC score for each model shows that logistic regression is the best performing classifier and more ‘stable’ model with 0.769 and 0.751 AUC score for BraTS and NSCLC, respectively.
• Our analysis on features highlights that *shape-based* and *gray level contrast* features are best performing features for MRI scanned data. While *grey level symmetry* features perform best for CT scanned images.
• Experiments and analysis show that logistic regression and linear regression models are more suitable models for survival prediction purposes as compared to decision tree, multilayer perceptron, artificial neural network, random forest, and support vector machine models.

The rest of the article is structured as follows: we explain the literature review in ‘Literature review’. Next, the details of the datasets are presented in ‘Dataset’. In ‘Methodology’, the proposed methodology to understand the medical images is detailed while the results and findings are explained in ‘Results’. Finally, we conclude our article in ‘Conclusion’.

## Literature review

In recent years, the research community has worked effectively on examining the medical images for survival prediction of patients (Kickingereder et al., 2016). For instance, Baid et al. (2018) and Baid et al. (2020) experimented with radiomic features of first-order statistics, shape-based 2D, shape-based 3D, gray level run length matrix (GLRLM), and GLCM using multilayer perceptron (MLP) and random forest models for survival prediction of brain tumor patients (Baid et al., 2018; Baid et al., 2020). Similarly, a study evaluated the performance of first-order statistics, GLCM, and neighbouring gray tone difference matric (NGTDM) along with age by applying a recursive feature selection method to select best performing features (Alam et al., 2018). Using the Extreme Gradient Boosting model, authors reported the age as most important feature for survival prediction. In addition, radiomic features like volumetric, textural, and shape-based were tested for survival prediction task of BraTS datasets (Baid et al., 2020). In a similar vein, pre-trained neural network model of the Visual Geometry Group (VGG16) was augmented with volumetric and age features for survival prediction of patient (Cabezas et al., 2018). Moreover, hand-crafted features such as tumor volume, volume ratio, surface area, the position of the enhancing tumor etc. have shown significant results for survival prediction of patients (Guo et al., 2019).

Selection of important features reducing the complexity and redundancy of features enhances the performance of machine learning models. In this regard, Gates et al. (2018) applied step-wise, univariate, and multivariate feature selection methods for selection of best radiomic features. Their analysis highlighted that gray level run length matrices (GLRLM) and NGTDM were not important features for patient survival prediction of brain tumor patients. In addition, the SVM model was also applied along with recursive feature elimination with cross-validation (RFECV). Moreover, location-based features of tumorous areas were tested for survival prediction (Soltani et al., 2021). In the study, authors compared the performance of LR, Support Vector Regression (SVR), and random forest (RF) regression models and concluded that tumor location-based features significantly impacted the performance of survival prediction task. Finally, the neuromorphic convolution neural network was also designed for survival prediction for brain tumor patients (Han & Han, 2018). Also, the performance of all radiomic features with feature selection methods of RFE, univariate feature selection, and *SelectFromModel* (Scikit-learn developers, 2021b) was evaluated. In a similar study (Sun & Zhang, 2018), authors extracted radiomic features and applied least absolute shrinkage and selection operator (LASSO) (Tibshirani, 1996) methods for selection of top features. In addition, researchers applied step-wise forward and backward selection methods for the selection of the top features (Hoerl & Kennard, 1970). For the experiment, MLP, RF, and support vector regression models were applied for predicting the survival days.

The survival prediction of lung cancer patients using CT scanned images and radiomic features is a well-explored area of research. Authors extracted radiomic and automatic features from CT scanned images using the ResNet18 network and used a CNN for the lung cancer patient survival time prediction (Ayyachamy et al., 2019). In addition, the binary classification (dead or alive) was done by utilizing radiomic features from CT scan features, clinical information, and ANN model (Chufal et al., 2019). The comparative analysis of the Bayesian network and SVM was also conducted for the survival prediction of patients (Jayasurya et al., 2010). The comparison highlighted the better performance of the Bayesian network with noisy data. Additionally, a study focusing on shape-based, intensity, and texture features of CT scanned images achieved the best performance (confidence interval 0.62−0.74) with gradient boosting linear model (Sun et al., 2018).

Table 1 shows the comparison of our work with the literature review. From this related work, we infer that researchers have used pre-selected classes of radiomic and hand-crafted features for survival prediction tasks. Authors use pre-selected three or four classes of radiomic features or hand-crafted features for the survival prediction task. Leveraging the literature, we perform a detailed comprehensive analysis of feature extraction, feature selection, and model selection methods to understand the medical images for the survival prediction task.

**Table 1:** Comparison of our work with literature.

**Sr#**	**Article**	**Feature class**								**Models** **used**	**Accuracy**
		**First order**	**Shape based**	**GLCM**	**GLSZM**	**GLRLM**	**NGTDM**	**GLDM**	**Hand crafted**		
**1**	Soltani et al. (Soltani et al., 2021)	Yes	Yes	Yes	Yes	Yes	Yes	Yes	No	LR, RFR, RFC, ANN	N/A
**2**	Baid et al. (Baid et al., 2020)	Yes	Yes	Yes	No	No	No	No	No	NN using MLP, RF	58.49%
**3**	Guo et al. (Guo et al., 2019)	No	No	No	No	No	No	No	Yes	Gradient Boosted Decision Tree	52%
**4**	Baid et al. (Baid et al., 2018)	Yes	Yes	Yes	No	Yes	No	No	No	MLP	57.1%
**5**	Alam et al. (Alam et al., 2018)	Yes	Yes	No	No	No	No	No	No	CNN	37%
**6**	Gates et al. (Gates et al., 2018)	Yes	No	Yes	No	Yes	Yes	No	No	RF	52
**7**	Sun et al. (Sun & Zhang, 2018)	Yes	Yes	No	No	No	No	No	No	Ridge Regression	N/A
8	Proposed Work	Yes	Yes	Yes	Yes	Yes	Yes	Yes	Yes	LoR, LR, MLPC, RFC, RFR, MLPR, SVM, ANN DTC, DTR,	66%

## Dataset

We use two datasets containing MRI and CT scanned images of brain tumor and lung cancer to develop models for survival prediction of cancer patients. Particularly, we use the BraTS 2020 (Menze et al., 2014; Bakas et al., 2017; Bakas et al., 2019) and NSCLC Radiomics (Aerts et al., 2014; Clark et al., 2013; Aerts et al., 2019) datasets for this research work. Table 2 describes the list of abbreviations used in the article.

**Table 2:** List of abbreviations and acronyms used in the article.

**SR#**	**Abbreviation**	**Explanation**	**SR#**	**Abbreviation**	**Explanation**
**1**	LR	Linear Regression	**25**	RFE	Recursive Feature Elimination
**2**	LoR	Logistic Regression	**26**	DICOM	Digital Imaging and Communications in Medicine
**3**	MLPC	Multilayer Perceptron Classifier	**27**	TesA	Testing Accuracy
**4**	MLPR	Multilayer Perceptron Regressor	**28**	MAE	Mean Absolute Error
**5**	RFC	Random Forest Classifier	**29**	RSME	Root Square Mean Error
**6**	RFR	Random Forest Regressor	**30**	SR#	Serial number
**7**	SVM	Support Vector Machine	**31**	FSM	Feature selection methods
**8**	EM	Evaluation Metrics	**32**	FS	Features Selected
**9**	DTC	Decision Tree Classifier	**33**	ANN	Artificial Neural Network
**10**	DTR	Decision Tree Regressor	**34**	MIr	Mutual_Info_regression
**11**	MIc	Mutual_Info_classification	**35**	Fr	F-test_regression
**12**	Fc	F-test_classification	**36**	ML	Machine learning
**13**	MinA	Minimum accuracy	**37**	MaxA	Maximum accuracy
**14**	AD	Accuracy difference	**38**	CT	Computed Tomography
**15**	MRI	Magnetic resonance imaging	**39**	NSCLC	Non-Small Cell Lung Cancer
**16**	GRACE	Global Registry of Acute Coronary Events	**40**	TIMI	Thrombolysis In Myocardial Infarction
**17**	APACHE-II	Acute Physiology And Chronic Health Evaluation II	**41**	NSTEMI	Non-ST-segment Elevation Myocardial Infarction
**18**	PET	Positron Emission Tomography	**42**	CADx	Computer-Aided Diagnosis
**19**	AI	Artificial intelligence	**43**	COVID-19	Coronavirus disease of 2019
**20**	GLRLM	Gray Level Run Length Matrix	**44**	PBS	Pitt Bacteremia Score
**21**	GLCM	Gray Level Co-occurrence Matrix	**45**	NGTDM	Neighbouring Gray Tone Difference Matric
**22**	RFECV	Recursive Feature Elimination with Cross-Validation	**46**	LASSO	Least Absolute Shrinkage and Selection Operator
**23**	AD	Accuracy difference	**47**	GLSZM	Gray Level Size Zone Matrix
**24**	AIC	Akaike Information Criterion	**48**	AUC	Area Under Curve

To predict the survival time of patients (brain tumor), we use 236 patients’ magnetic resonance images (MRI) from the BraTS dataset released in 2020. BraTS dataset is released on yearly basis and from 2017 onwards, BraTS is providing information on the survival days of patients along with MRI (Bakas et al., 2017). BraTS contains native (T1), T2-weighted (T2), post-contrast T1-weighted (T1Gd), and T2 Fluid Attenuated Inversion Recovery (T2-FLAIR) volume images of each patient extracted by varying clinical conventions and scanners (Bakas et al., 2017). Also, the ground truth image containing labels of four classes of enhancing tumor (tumor), edema (ED), necrosis and non-enhancing tumor (NCR/NET), and “other” is provided. In addition, the overall survival of each patient is available in days. Each MRI and ground truth image is compressed to neuroimaging informatics technology initiative (NIfTI) records and consists of a 3D image of 240 × 240 × 155 pixels.

We also use the NSCLC dataset containing CT scanned images of lung cancer patients. The dataset contains records of 422 patients and all CT scanned images are accessible as digital imaging and communications in medicine (DICOM) records. There are a series of CT scans available for each patient. In addition, one Segmentation and Radiotherapy Structure Sets DICOM image for each patient as ground truth is available. Each Segmentation and Radiotherapy Structure Sets image contains a manual outline of the 3D volume of the essential gross tumor volume (GTV-1) by a radiation oncologist. Also, gender, overall stage, and death status of patients along with a patient survival time are available as additional clinical info. Please note that BraTS and NSCLC provide anonymized data which limits the acquisition of censoring information of patients. However, standardization and benchmarking of both datasets inherit that patients are observed for the longest possible bucket of time which nullifies the impact of censoring on survival prediction. For instance, BraTs and NSCLC datasets report survival time of patients upto 1,767 days (4.48 years) and 4454 days (12.2 years), respectively.

For patient survival time prediction, the number of days of each patient is provided for each training sample of BraTS and NSCLC dataset. Despite the quantitative labels of overall patient survival time, the BraTS overall patient survival prediction task handles it as a classification task by binning the patient survival days into three bins. We label the patient survival days as 0, 1, and 2 if the survival days fall into the range of 0–300, 300–450, and >450 days, respectively as done by BraTS. Similar binning is applied for the NSCLC dataset as well. Additionally, we randomly split each dataset into training and testing datasets with the ratio of 80:20 respectively. Overall statistics of datasets are given in Table 3.

## Methodology

In this section, first, we describe feature extraction and selection methods chosen for the study. Next, we present the machine learning models and evaluation metrics used to benchmark the image features. Finally, we dive into the experimental setup of our article.

### Feature extraction and selection

**Figure 1:**
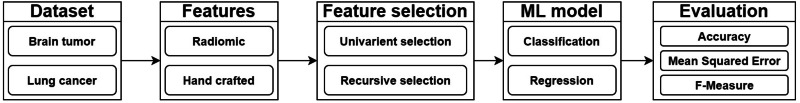
Flow diagram of our methodology to understand medical images for survival prediction.

Figure 1 shows the basic flow diagram of our proposed methodology. Our first chosen feature extraction method is *radiomic* features. Radiomic features extract sub-visual and quantifiable data from the images which can not be obtained from the naked eye. For in-depth analysis, we further categorize radiomic features into 8 classes in accordance with Van Griethuysen et al. (2017). Precisely, radiomic features are categorized into first order, shape based 2D, shape based 3D, Gray-Level Co-Occurrence Matrix (GLCM), Gray Level Run Length Matrix (GLRLM), Gray Level Size Zone Matrix (GLSZM), Neighborhood Grey Tone Difference Matrix (NGTDM), and Gray Level Dependence Matrix (GLDM) features. Table 4 shows the statistics of radiomic feature classes and few example features of every class. First order statistics examines the intensity of pixels in images using standard metrics like energy, 10th percentile, minimum, and median values. For instance, energy of image pixels is calculated using Eq. (1).

(1) \begin{eqnarray*}E=\sum _{i=1}^{{N}_{p}}(X(i)+c)^{2}\end{eqnarray*}

where *X* is the set of *N*_*p*_ number of voxels within the ROI and *c* is optional parameter.

**Table 3:** Statistics of BraTS 2020 and NSCLC radiomics dataset.

**SR#**	**Dataset**	**Images**			**Image type**	**Image size**	**Disease type**	**#of patients**	**Survival Info**		
		Train	Test	Total					Train	Test	Total
**1**	BraTS 2020	369	125	494	MRI	240 ×240 ×155	Brain Tumor	494	189	47	236
**2**	NSCLSC Radiomics	338	84	422	CT Scan	512 ×512 ×(75–297)	Lung cancer	422	338	84	422

**Table 4:** Statistics of feature classes and example features.

**SR#**	**Feature class**	**# of Features**	**Example**
**1**	First Order Statistics	19	Energy, 10th Percentile, Median, Mean
**2**	Shape Based 3D	16	Mesh volume, Surface area, Compactness, Elongation
**3**	Shape Based 2D	10	Sphericity, Major axis length, Perimeter
**4**	GLCM	24	Joint energy, Joint average, Sum variance, Sum entropy
**5**	GLSZM	16	Small area emphasis, Gray level non-uniformity
**6**	GLRLM	16	Gray level variance, Run percentage, Run variance
**7**	NGTDM	5	Coarseness feature value, Complexity feature value
**8**	GLDM	14	Low gray level emphasis, Gray level variance
**9**	Hand Crafted	36	Brain tumor core x, Whole SV ratio, Edema grad
**10**	Total	156	

Shape-based features *i.e.,* volume of voxel, surface area, and area to volume ratio describe the size and shape of tumorous area in the image. For example, the sphericity of 3D image with the volume (*V*) and area (*A*) is computed using Eq. (2).

(2) \begin{eqnarray*}sphericity= \frac{\sqrt[3]{36\pi {V}^{2}}}{A} .\end{eqnarray*}

Sphericity measures the roundness of objects in the image. Similarly, compactness, axis lengths, flatness, and elongation extract the features to understand the shape of tumor in images.

Gray Level Co-occurrence Matrix (GLCM) uses masks to calculate the distance between similar pixel values to extract the correlation in different regions of the image. Like, Cluster Prominence (CP) measures the skewness and symmetry of an image while Inverse Difference Moment (IDM) estimates the local homogeneity. CP and IDM are computed using Eqs. (3) and (4), respectively.

(3) \begin{eqnarray*}\text{CP}=\sum _{a=1}^{{N}_{g}}\sum _{b=1}^{{N}_{g}}(a+b-{u}_{x}-{u}_{y})^{4}p(a,b)\end{eqnarray*}

(4) \begin{eqnarray*}\text{IDM}=\sum _{a=0}^{{N}_{g}-1} \frac{{{p{}_{x}}_{-}}_{y}(a)}{1+{a}^{2}} \end{eqnarray*}

where *u*_*x*_ and *u*_*y*_ represents mean grey level intensities of x and y dimension while *p*(*a*, *b*) is normalized value of co-occurrence matrix. Also, *p*_*x*_ represents the marginal row probability. Likewise, the variants of standard metrics like cluster share, contrast, entropy variance, joint entropy etc. are calculated for GLCM features. Gray Level Run Length Matrix (GLRLM) aggrandizes the GLCM by mapping the consecutive pixels that have the same gray level value. As an example, Gray Level Non-Uniformity (GLN) measures the similarity of gray-level intensity values in the image. Also, the dependency emphasis metrics examine the texture of image by estimating the distribution of pixels dependence. GLN and Short-Run Emphasis (SRE) are calculated using Eqs. (5) and (6), respectively.

(5) \begin{eqnarray*}\text{GLN}= \frac{{\mathop{\sum \nolimits }\nolimits }_{i=1}^{{N}_{g}}({\mathop{\sum \nolimits }\nolimits }_{j=1}^{{N}_{t}}P(i,j{|}\theta ))^{2}}{{N}_{r}(\theta )} \end{eqnarray*}

(6) \begin{eqnarray*}\text{SRE}= \frac{{\mathop{\sum \nolimits }\nolimits }_{i=1}^{{N}_{g}}{\mathop{\sum \nolimits }\nolimits }_{b=1}^{{N}_{d}} \frac{P(i,j)}{{j}^{2}} }{{N}_{z}} \end{eqnarray*}

where *N*_*r*_(*θ*) is the number of runs in the image along angle *θ*. Like GLRLM, Gray Level Size Zone Matrix (GLSZM) quantifies gray level zones in the connected pixel zones. Similar metrics such as Small Area Emphasis (SAE), Gray Level Non-Uniformity Normalized (GLNN), and Size-Zone Non-Uniformity (SZN) are extracted as GLSZM features.

Neighbouring Gray Tone Difference Matrix (NGTDM) measures the difference between grey level value of center and average value of neighbours. For example, coarseness indicates the spatial rate of change using Eq. (7)

(7) \begin{eqnarray*}\text{Coarseness}= \frac{1}{\sum _{i=1}^{{N}_{g}}{p}_{i}{s}_{i}} \end{eqnarray*}

where *s*_*i*_ sum of absolute differences for gray level *i*. Finally, Gray Level Dependence Matrix (GLDM) excerpts grey level dependencies in an image. Precisely, dependency is calculated by measuring pixels connected and dependent to center pixel of image. Features such as Gray Level Variance (GLV), Dependence Entropy (DE), and Low Gray Level Emphasis (LGLE) are derived from variance, entropy, and gray level emphasis metrics, respectively.

In addition, we notice that researchers have experimented with hand-crafted features to analyze the BraTS dataset. Leveraging these features (Guo et al., 2019), we test 36 hand-crafted features like tumor volume, volume ratio, surface area, position of the enhancing tumor etc. for survival prediction. Overall, we test the performance of 156 radiomic and hand-crafted features for the survival prediction of cancer patients.

Feature selection is a process to improve the performance of machine learning models by selecting the most contributing features only (Kira & Rendell, 1992). Therefore, we test the performance of univariate and recursive feature elimination methods. Univariate methods perform a univariate measurable test on features to measure their relationship for selection. Precisely, we choose F-test and mutual information method from the univariate feature selection category. Similarly, recursive feature elimination methods recursively remove the redundant and negligible features after training and analyzing the performance of machine learning models. We use recursive features elimination (RFE) and recursive feature elimination with cross-validation (RFECV) methods for survival prediction. In addition, univariate and recursive methods are tested for both classification and regression tasks.

### Machine learning models and evaluation

The scope of our research is to test the performance of supervised machine learning models in the context of survival prediction. In this regard, we choose Decision Tree Classifier (DTC) (Safavian & Landgrebe, 1991), Support Vector Machine (SVM) (Noble, 2006), Logistic Regression (LoR) (Kleinbaum et al., 2002), and Random Forest Classifier (RFC) for classification of survival time. In addition, we test the neural network models of Multilayer Perceptron Classifier (MLPC) (Gardner & Dorling, 1998) and Artificial Neural Network (ANN) (Hassoun et al., 1995). MLP are strictly feed forward neural networks while ANN models can contain loops (Simplilearn, 2022). Similarly, state-of-the-art models of Multilayer Perceptron Regressor (MLPR), Decision Tree Regressor (DTR) Linear Regression (LR) (Montgomery, Peck & Vining, 2012), and Random Forest Regressor (RFR) (Pal, 2005) are tested for regression task. For the evaluation of models, we select standard evaluation metrics. For instance, classification performance is tested using measures of accuracy and Area Under Curve (AUC) (Goutte & Gaussier, 2005; Story & Congalton, 1986). Accuracy is simply the ratio between correctly predicted survival days of patients and the total number of survival patients. Moreover, AUC utilizes the specificity and sensitivity of the model to measure the ability of a classifier to distinguish between classes. Similarly, the regression models are tested using accuracy, Mean Absolute Error (MAE), Root Mean Squared Error (RMSE), and Akaike information criterion (AIC). Absolute Error is a difference between predicted patient survival days and actual patient survival days. The average of absolute errors of all samples is named Mean Absolute Error (MAE). The square root of the average absolute error is called root Mean Squared Error (RMSE). Additionally, AIC is a statistical measure which uses log-likelihood to estimate the quality of trained model.

### Experimental setup

We create combinations of different feature classes, feature selection methods, and machine learning models. Our two defined feature classes are radiomic and hand-crafted features. Also, we further merge six grey level features from radiomic features due to their efficacy to create ‘grey level feature’. Precisely, we combine and test GLCM, GLDM, GLRLM, GLSZM, and NGTDM features. Also, we combine the first order and shape-based features. It is pertinent to note that we also merge shape-based 3D and 2D features as ‘shape-based’ features. Moreover, we augment these features with hand-crafted features. For evaluation of these eight feature classes, we formulate 540 combinations of feature classes, six feature selection methods, and ten models. All possible combinations of features, selection methods, and machine learning models are tested. Table 5 shows the sample combinations formulated for evaluation. Furthermore, we train our model using python 3.9 with Anaconda package management installed on Windows 10. We use the default parameters for machine learning models for training.

**Table 5:** Example of formulated combinations.

**SR#**	**Feature class**	**FSM**	**Model**
1	First order	Fc	LoR
2	Shape based	MIc	SVM
3	GLCM	MIc	MLPC
4	Radiomic features	MIc	SVM
5	GLRLM	Fr	DTC
6	First order	MIr	SVM
7	NGTDM	MIc	RFC
8	Hand crafted features	MIc	SVM

## Results

In this section, first, we describe our analysis of the BraTS dataset. Next, the evaluation results of the NSCLC dataset are presented. Finally, we select the best models for survival prediction of patients by analyzing the “*stability*” of models.

### BraTS dataset

We initiate our analysis by extracting the *base results* of all feature classes without applying feature selection methods. Please note that we adopt five fold cross validation approach to test all models by varying training and testing data distribution (Refaeilzadeh, Tang & Liu, 2009; Nawaz, Khan & Qadri, 2022; Chatterjee et al., 2022; Xu et al., 2021). Tables 6 and 7 shows the result of BraTS dataset for classification and regression models, respectively. We provide the *base result* and the highest improvement achieved by models after applying feature selection method. Moreover, best performing feature selection method (FSM) and number of top features (FS) selected are also given. Focusing on classification results, we notice that NGTDM features outperform with 63% accuracy using the RF classifier. Also, logistic regression models failed to perform using all features with 48.2% average accuracy. The poor performance of the LoR model is attributed to the redundancy of features (King & Zeng, 2001). Also, the hand-crafted and first-order features perform poorly with up to 47% and 42% accuracy, respectively. Moreover, despite the low accuracy of the DT classifier with all features, the classifier achieves 52% accuracy with GLCM features. The reason is that in the case of GLCM features the classifier learns better rules to predict test data. On the other hand, we observe that generally, RFC models achieve > 49% accuracy except for hand-crafted and shape-based feature classes. In the case of regression, we achieve the highest 52% accuracy using linear regression and MLP models. However, the Root Mean Squared Error (RMSE) of the MLP model is very large highlighting the poor generalizability of the model due to overfitting. In addition, our analysis on a combination of features highlights that combination of first-order and shape-based features achieves 31% accuracy with LoR model. Also, adding the handcrafted features in this combination improves the accuracy by 17%. While the linear regression model shows 37–46% accuracy with the same combination of features. Moreover, maximum 50% accuracy is achieved with the combination of all grey-level features and LoR model. Results also highlight that classification and regression models did not show a significant improvement in results.

**Table 6:** Results of classification models on BraTS 2020 dataset.

**SR#**	**Feature class**	**EM**	**LoR**	**MLPC**	**SVM**	**RFC**	**DTC**	**ANN**
**1**	First Order	TesA	0.3+0.35	0.14+0.43	0.49+0.05	0.52+0.15	0.39+0.16	0.52+0.02
		FSM	Fc	MIc	Fc	Fc	Fr	MIc
		FS	16	10	6	12	2	7
**2**	Shape Based	TesA	0.42+0.22	0.22+0.34	0.37+0.22	0.45+0.09	0.37+0.1	0.48+0.15
		FSM	Fc	Fc	MIr	Fc	Fr	Fr
		FS	2	6	3	all	2	7
**3**	GLCM	TesA	0.48+0.07	0.27+0.31	0.41+0.13	0.59+0.03	0.52+0.1	0.52+0.06
		FSM	Fc	Fr	Fc	MIc	Fr	Fc
		FS	2	6	9	17	13	9
**4**	GLDM	TesA	0.5+0.1	0.51+0.07	0.3+0.28	0.47+0.15	0.33+0.2	0.52+0.06
		FSM	Fc	MIr	MIc	Fr	MIr	Fr
		FS	2	5	8	6	3	9
**5**	GLRLM	TesA	0.57+0.09	0.26+0.4	0.11+0.48	0.54+0.09	0.29+0.27	0.53+0.1
		FSM	MIr	MIc	Fr	Fr	MIr	MIc
		FS	5	6	11	8	6	11
**6**	GLSZM	TesA	0.48+0.08	0.34+0.3	0.29+0.27	0.52+0.13	0.45+0.13	0.52+0.11
		FSM	Fr	MIc	MIc	Fr	MIr	Fc
		FS	3	12	3	6	6	3
**7**	NGTDM	TesA	0.54+0.03	0.4+0.14	0.45+0.08	0.6+0.03	0.4+0.08	0.49+0.06
		FSM	Fc	Fr	Fr	Fc	Fc	MIc
		FS	2	5	all	4	2	2
**8**	Radiomic	TesA	0.43+0.17	0.14+0.46	0.14+0.52	0.49+0.17	0.33+0.17	0.5+0.16
		FSM	Fc	Fr	MIc	MIc	Fc	Fr
		FS	2	2	22	all	6	16
**9**	Hand Crafted	TesA	0.42+0.07	0.34+0.25	0.3+0.23	0.47+0.1	0.35+0.18	0.46+0.09
		FSM	MIr	MIr	MIr	MIr	Fc	MIc
		FS	2	7	9	21	6	6
**10**	First Order +Shape Based	TesA	0.31+0.34	0.13+0.43	0.14+0.43	0.51+0.11	0.4+0.06	0.49+0.08
		FSM	Fc	Fr	MIc	MIr	Fc	Fr
		FS	2	10	17	17	16	14
**11**	GLCM+GLDM +GLRLM+GLSZM + NGTDM	TesA	0.5+0.14	0.49+0.12	0.24+0.41	0.53+0.08	0.37+0.15	0.57+0.06
		FSM	Fc	MIr	MIr	Fc	Fr	Fc
		FS	3	20	13	22	15	13
**12**	Radiomic +Hand Crafted	TesA	0.48+0.12	0.49+0.04	0.16+0.39	0.53-0.02	0.29+0.18	0.5+0.03
		FSM	Fc	MIc	MIr	Fc	MIr	Fc
		FS	14	4	12	19	11	3
**13**	First Order +Shape Based +Hand Crafted	TesA	0.5+0.08	0.31+0.23	0.51+0.06	0.4+0.21	0.34+0.14	0.5+0.06
		FSM	Fr	MIr	Fr	Fr	Fr	Fc
		FS	16	2	12	21	16	16

**Table 7:** Regression models results on BraTS 2020 dataset.

**SR#**	**Feature class**	**EM**	**LR**	**MLPR**	**RFR**	**DTR**
**1**	First Order	TesA	0.43+0.04	0.32+0.2	0.32+0.14	0.41+0.17
		MAE	231.64	414.74	243.66	258.35
		RMSE	294.48	519.32	328.51	401.63
		AIC, *p*-value	−10.90 0.0011	433.62, 0.0011	−7.53, 0.0001	34.39 0.0002
		FSM, FS	MIr, 15	Fc, 14	MIr,6	MIr, 15
**2**	Shape Based	TesA	0.38+0.1	0.29+0.25	0.38+0.14	0.42+0.16
		MAE	206.57	795.47	233.61	345.77
		RMSE	268.76	913.05	336.14	487.8
		AIC, *p*-value	−2869.70, 0.0007	278.69 0.0001	202.85 0.0003	119.80,0.0024
		FSM, FS	Fc, 3	MIr, 10	Fr, 2	Fr, 2
**3**	GLCM	TesA	0.4+0.08	0.52+0.07	0.36+0.1	0.44+0.09
		MAE	224.04	250.19	234.17	344.6
		RMSE	293.36	374.04	327	496.8
		AIC, *p*-value	−3.95, 0.0036	371.74, 0.0003	−0.73, 0.0001	23.41, 0.0002
		FSM,FS	Fr,18	Fc,16	Fr,5	Fr,5
**4**	GLDM	TesA	0.33+0.07	0.3+0.23	0.38+0.1	0.39+0.14
		MAE	225.69	325.88	252.74	313.94
		RMSE	281.74	442.39	332.64	453.17
		AIC, *p*-value	−5.88 0.0003	11.78 0.0015	−5.67, 0.0011	22.71 0.0005
		FSM,FS	Fr,2	MIr,3	Fr,2	Fr,3
**5**	GLRLM	TesA	0.39+0.07	0.27+0.25	0.48+0.08	0.44+0.09
		MAE	232.51	366.56	220.76	338.31
		RMSE	287.39	469.85	292.38	500.58
		AIC, *p*-value	−6.86, 0.0004	222.75, 0.0006	−10.87, 0.0007	16.71, 0.0002
		FSM,FS	Fr,4	Fc,2	Fr,4	Fc,3
**6**	GLSZM	TesA	0.42+0.06	0.27+0.24	0.44+0.08	0.38+0.19
		MAE	264.95	367.6	232.35	295.58
		RMSE	381.33	470.67	305.39	453.94
		AIC, *p*-value	0.032, 0.0021	1267.67, 0.0007	−7.76, 0.0012	16.71, 0.0002
		FSM,FS	MIr,12	Fc,2	Fc,14	Fr,6
**7**	NGTDM	TesA	0.4+0.03	0.25+0.3	0.44+0.05	0.37+0.18
		MAE	227.36	235.77	235.68	288.25
		RMSE	293.97	351.65	316.5	441.41
		AIC, *p*-value	−3.13, 0.0008	15.89, 0.0002	−9.21, 0.0002	15.08, 0.0004
		FSM,FS	Fc,5	MIr,5	Fr,5	MIr,5
**8**	Radiomic	TesA	0.49+0.03	0.51+0.03	0.32+0.21	0.37+0.2
		MAE	552.94	3227.4	222.29	275.33
		RMSE	1013.36	8417.41	274.38	394.79
		AIC, *p*-value	−71.18, 0.0017	202.14, 0.0017	−11.61, 0.0002	26.12, 0.0010
		FSM,FS	Fc,all	MIr,19	Fr,5	MIr,7
**9**	Hand Crafted	TesA	0.38+0.09	0.41+0.09	0.27+0.2	0.23+0.25
		MAE	227.32	367.31	219.73	305.13
		RMSE	306.02	472.31	300.86	425.47
		AIC, *p*-value	−1.067, 0.0005	667.62, 0.0011	−0.57, 0.0005	24.04, 0.0003
		FSM,FS	Fc,19	Fc,2	Fc,9	MIr ,21
**10**	First Order +Shape Based	TesA	0.44+0.06	0.52+0.0	0.41+0.15	0.41+0.14
		MAE	220.26	361.01	217.59	241.02
		RMSE	281.62	465.08	281.89	314.71
		AIC, *p*-value	0.22, 0.0003	125.87, 0.0014	−6.67, 0.0004	30.51, 0.0003
		FSMF,FS	Fc,13	Fc,2	Fr,4	Fc,21
**11**	GLCM+GLDM +GLRLM+GLSZM +NGTDM	TesA	0.44+0.02	0.49+0.07	0.4+0.1	0.4+0.13
		MAE	236.25	315.92	230.57	281.65
		RMSE	294.58	450.55	298.25	390.29
		AIC, *p*-value	−6.67, 0.0006	1084.23, 0.0007	−10.07, 0.0002	22.07, 0.0002
		FSM,FS	Fr,5	Fr,17	Fr,2	Fr,5
**12**	Radiomic +Hand Crafted	TesA	0.49+0.01	0.49+0.03	0.23+0.2	0.4+0.17
		MAE	527.28	368.01	238.3	355.77
		RMSE	1118.68	476.19	316.04	539.88
		AIC, *p*-value	63.21 0.0014	641.72 0.0006	−4.68 0.0013	35.86 0.0003
		FSM,FS	Fc,all	Fc,9	Fr,21	MIr,16
**13**	First Order +Shape Based +Hand Crafted	TesA	0.38+0.07	0.49+0.01	0.27+0.16	0.49+0.1
		MAE	236.24	368.98	281.17	252.02
		RMSE	301.57	473.64	389.32	386.22
		AIC, *p*-value	−3.47, 0.003	389.87, 0.0020	2.04, 0.0002	23.23, 0.0001
		FSM,FS	Fr,11	Fc,2	Fr,2	MIc,7

Next, we focus on analyzing the performance of machine learning models with *top features* selected by six feature selection methods. For an extensive analysis of features, the performance of models is evaluated by training the models with top features ranging from two to ‘all’. In this scenario, ‘all’ features represent the base results of the feature class. We observe that the performance of the logistic regression classifier improves from 42% to 64% accuracy as compared to base results with a selection of top two features with the Fc feature selection method. The improvement of 22% accuracy is attributed to the removal of redundant and insignificant from training and testing data. Moreover, the NGTDM class highlights that all features of the class are important for machine learning models and we get the best 54% accuracy with the SVM classifier. In addition, our results depict that selection of only seven hand-crafted features shows an improvement of 25% accuracy.

Our manual analysis of best-performing features infers that age is the most important feature in the prediction of patient survival days. Moreover, results highlight that classification feature selection methods of Fc and MIc outperform other feature selection methods. In general, we conclude that the logistic regression model achieves the best accuracy of 66% with the top five features selected from GLRLM class with the MIr feature selection method. In addition, feature selection methods improve the performance of classification and regression models by 22% and 10% accuracy, respectively. This result infers that classification models are significantly impacted by feature selection methods.

### NSCLC dataset

Tables 8 and 9 show base results of classifier and regression models on the NSCLC dataset. Contrary to the BraTS dataset, results show that LoR and LR models achieve >49% accuracy for each feature class. Also, the ANN model shows more than 49% accuracy. The testing shows that the ANN model is not generalizable because the value of accuracy varies from 33% to 68% for each distribution of the dataset. Also, SVM, MLPC, and MLPR models failed to perform because the results of these models vary by running each model multiple times due to the changing distribution of data.

**Table 8:** Results of classification models on NSCLC dataset.

**SR#**	**Feature class**	**EM**	**LoR**	**MLPC**	**SVM**	**RFC**	**DTC**	**ANN**
**1**	First Order	TesA	0.5+0.0	0.32+0.22	0.5+0.02	0.47+0.08	0.42+0.08	0.49+0.01
		FSM	Fc	MIc	Fc	MIr	MIr	MIr
		FS	2	5	3	4	2	4
**2**	Shape Based	TesA	0.5+0.03	0.52+0.03	0.32+0.2	0.52+0.01	0.49+0.08	0.48+0.04
		FSM	MIc	MIr	Fr	MIr	MIr	MIc
		FS	8	6	11	2	2	4
**3**	GLCM	TesA	0.51+0.01	0.32+0.22	0.53-0.01	0.48+0.08	0.38+0.11	0.49+0.02
		FSM	Fc	Fc	MIr	MIr	Fr	MIr
		FS	4	4	14	11	5	19
**4**	GLDM	TesA	0.5+0.02	0.34+0.16	0.26+0.26	0.46+0.1	0.37+0.18	0.48+0.05
		FSM	MIr	MIr	MIr	MIr	MIc	Fc
		FS	10	4	4	2	2	12
**5**	GLRLM	TesA	0.53+0.04	0.44+0.08	0.37+0.15	0.44+0.09	0.34+0.15	0.47+0.03
		FSM	MIr	Fr	Fc	MIc	MIr	MIr
		FS	6	4	4	2	14	13
**6**	GLSZM	TesA	0.5+0.02	0.36+0.15	0.35+0.15	0.46+0.06	0.43+0.08	0.49+0.0
		FSM	Fc	Fc	Fc	MIr	MIc	Fc
		FS	2	2	2	4	4	2
**7**	NGTDM	TesA	0.52+0.02	0.47+0.07	0.32+0.22	0.5+0.07	0.41+0.17	0.49+0.03
		FSM	Fc	MIr	MIc	MIr	MIr	MIc
		FS	all	all	all	3	5	2
**8**	Radiomic	TesA	0.51+0.03	0.51+0.03	0.49+0.02	0.5+0.04	0.41+0.09	0.5+0.01
		FSM	Fc	MIc	MIr	MIr	MIc	Fc
		FS	6	2	3	16	11	14
**9**	First Order +Shape Based	TesA	0.51+0.03	0.5+0.04	0.52+0.01	0.5+0.08	0.42+0.09	0.51+0.02
		FSM	Fc	MIr	Fc	MIr	MIr	Fc
		FS	4	3	5	7	2	2
**10**	GLCM+GLDM +GLRLM+GLSZM +NGTDM	TesA	0.51+0.02	0.5+0.04	0.38+0.15	0.47+0.07	0.41+0.06	0.48+0.05
		FSM	Fc	MIc	MIc	MIr,MIc	Fr	
		FS	13	2	3	17	22	13
**11**	Shape Based +GLRLM+NGTDM	TesA	0.49+0.05	0.3+0.24	0.48+0.02	0.48+0.06	0.43+0.16	0.5+0.04
		FSM	Fc	MIc	Fc	MIc	MIr	MIc
		FS	5	2	2	10	2	11

**Table 9:** Regression models results on NSCLC dataset.

**SR#**	**Feature class**	**EM**	**LR**	**MLPR**	**RFR**	**DTR**
**1**	First Order	TesA	0.49+0.03	0.35+0.2	0.49+0.05	0.47+0.19
		MAE	858.14	867.01	790.89	509.06
		RMSE	1203.85	1156.16	1031.55	919.16
		AIC, *p*-value	−29.55, 0.0021	−17.38, 0.0029	−27.41, 0.0017	65.26, 0.0007
		FSM,FS	Fr,10	Fc,3	MIc,8	MIr,2
**2**	Shape Based	TesA	0.47+0.04	0.48+0.04	0.5+0.09	0.4+0.19
		MAE	928.24	879.41	687.27	718.04
		RMSE	1298.5	1150.78	943.85	1108.15
		AIC, *p*-value	−22.89, 0.0012	−19.47, 0.0009	−29.14, 0.0003	74.33, 0.0006
		FSM,FS	Fc,13	MIr,2	MIr,2	MIr,3
**3**	GLCM	TesA	0.49+0.03	0.5+0.04	0.51+0.04	0.41+0.22
		MAE	938.67	830.72	937.02	593.93
		RMSE	1208.09	1132.43	1204.82	952.84
		AIC, *p*-value	−22.98, 0.0011	−30.77, 0.0009	−3.52, 0.0019	61.20, 0.0018
		FSM,FS	Fr,14	Fr,10	Fr,5	MIr,2
**4**	GLDM	TesA	0.51+0.0	0.49+0.05	0.52+0.04	0.48+0.13
		MAE	942.89	834.05	611.46	659.26
		RMSE	1107.04	1140.74	809.24	1104.38
		AIC, *p*-value	−26.52, 0.0031	321.19, 0.0014	−28.26, 0.0009	66.59, 0.0005
		FSM,FS	Fc,2	Fc,8	MIc,2	Fc,2
**5**	GLRLM	TesA	0.5+0.02	0.52+0.05	0.49+0.06	0.48+0.15
		MAE	865.31	846.52	730.63	883.05
		RMSE	1108.9	1148.12	948.39	1369.78
		AIC, *p*-value	−27.27, 0.0025	356.71, 0.0003	−11.23, 0.0021	84.64, 0.0003
		FSM,FS	Fc,2	Fc,10	MIr,4	Fc,5
**6**	GLSZM	TesA	0.49+0.02	0.33+0.22	0.47+0.06	0.44+0.13
		MAE	851.02	853.07	688.84	649.75
		RMSE	1104.88	1140.92	907.73	1122.23
		AIC, *p*-value	−28.89, 0.0027	−27.94, 0.0035	−16.65, 0.0015	86.97, 0.0009
		FSM,FS	MIr,3	Fc,2	MIc,2	Fc,2
**7**	NGTDM	TesA	0.51+0.03	0.54+0.01	0.5+0.03	0.47+0.12
		MAE	851.04	845.11	755.27	607.07
		RMSE	1102.23	1145.89	981.17	992.18
		AIC, *p*-value	−25.70,0.0012	23.39,0.0022	−35.74,0.0004	63.25,0.0010
		FSM,FS	Fc,2	Fc,3	MIc,3	Fc,2
**8**	Radiomic	TesA	0.51+0.0	0.32+0.22	0.5+0.05	0.42+0.18
		MAE	855.24	853.32	701.19	782.29
		RMSE	1100.16, 0.0028	1148.03, 0.0028	956.90, 0.0013	1169.21, 0.0003
		AIC, *p*-value	−25.34, 0.0028	−24.80, 0.0028	−15.96, 0.0013	71.16, 0.0003
		FSM,FS	Fc,2	Fc,2	MIr,2	MIc,6
**9**	First Order+ Shape Based	TesA	0.47+0.03	0.41+0.14	0.52+0.06	0.44+0.13
		MAE	855.24	863.81	700.87	736.83
		RMSE	1100.16	1152.2	950.9	1144.94
		AIC, *p*-value	−23.40, 0.0023	254.72, 0.0033	−30.68, 0.0011	59.82, 0.0002
		FSM,FS	Fc,2	Fc,3	MIr,2	MIr,3
**10**	GLCM+GLDM+ GLRLM+GLSZM +NGTDM	TesA	0.5+0.04	0.37+0.18	0.49+0.05	0.45+0.16
		MAE	868.84	839.42	773.03	785.29
		RMSE	1110.19	1134.89	1004.97	1221.86
		AIC, *p*-value	−25.34, 0.0027	−18.16, 0.0008	−16.38, 0.0004	57.72, 0.0040
		FSM,FS	Fc,2	MIr,2	MIr,4	MIc,2
**11**	Shape Based+ GLRLM+NGTDM	TesA	0.48+0.03	0.45+0.09	0.48+0.08	0.49+0.12
		MAE	853.73	853.76	691.05	758.28
		RMSE	1124.91	1140.34	951	1206.06
		AIC, *p*-value	−21.89, 0.0012	−26.80, 0.0005	4.70, 0.0007	76.80, 0.0002
		FSM,FS	MIr,17	Fc,2	MIr,2	MIc,2

Analyzing the individual feature class, the feature selection method MIr with GLRLM features and the LoR model achieve the highest accuracy of 57%. Moreover, the LoR model with the MIr feature selection method achieves the maximum improvement in accuracy by 0.08%. We also note that models with mutual information feature selection methods achieve better accuracy compared to F-test feature selection methods. The reason for the failure of the F-test feature methods is that they can handle only the linear dependency of features (Scikit-learn developers, 2021a). However, mutual information feature selection methods can handle various types of dependencies between features. Also, this result shows that BraTS has a more linear dependency on features compared to NSCLC. Focusing on a combination of feature classes, results show that the combination of shape-based, GLRLM and NGTDM achieves the highest accuracy of 59% and 61% for classification and regression, respectively.

Next, we shift our focus to comparing the performance of features for BraTS and NSCLC datasets. The comparison shows that grey level features outperform for the prediction of lung cancer patients in the NSCLC dataset. While shape-based and first-order features are among the best-performing features for the BraTS dataset. In addition, for the BraTS dataset, age is selected as the top feature by the best-performing combination of first-order and shape-based features using the LoR model with the Fc feature selection method. On contrary, age is listed as the 5th top feature by the mutual information feature selection method for NSCLC. Also, the logistic regression model shows better performance with >50% accuracy for both datasets. Similarly, the SVM classifier shows a 54% and 52% value of accuracy with GLCM features for BraTS and NSCLC, respectively. While MLP and decision tree regressor failed for both datasets with high root mean square error as shown in Tables 7 and 9.

### Stability analysis

Our extensive analysis of combinations of features, feature selection methods and machine learning models signifies the best performing combination. However, as mentioned earlier, the performance of models is varied by changing the distribution of training and testing data. This observation implies that models achieving the best accuracy are not generalized models as well. Hence, we didn’t rely only on the best accuracy of combination to select the final model. For the final selection of classification models, we calculate the *stability* and Area Under Curve (AUC) of best performing five combinations. We label the combination as stable which fulfils two conditions. First, the minimum and maximum accuracy of the combination did not vary after testing the model on different training and test sets drawn from the data. Second, the combination achieves the highest AUC value highlighting the confidence of the model to measure of separability of classes. In particular, we test each combination using five fold cross validation after varying training and testing data. Table 10 shows the results of testing stability of best-performing combinations for BraTS and NSCLC datasets for classification. The table provides values of feature selection method (FSM), number of top features selected (FS), minimum (MinA) and maximum accuracy (MaxA) achieved in three iterations, Difference between accuracy (AD), and AUC. We notice that LoR model along with its respective features outperforms for classification of NSCLC and BraTS datasets. The model shows zero variance in accuracy and attains the highest AUC values of 0.769 and 0.751 for BraTS and NSCLC, respectively.

Focusing on regression models, Table 11 presents the analysis for regression models for BraTS and NSCLC datasets. Similar to classification, we also rely on minimum change in accuracy to label the stable model. Also, we test Akaike Information Criterion (AIC) to identify the stable models with least AIC value (Sakamoto, Ishiguro & Kitagawa, 1986). The results indicate the superior performance of LR model with zero variance in accuracy. Moreover, these results are further substantiated by lower AIC values of −71.18 and −29.55 for BraTS and NSCLC, respectively. In addition, we manually analyze the top selected features of models. Table 12 shows the top selected features. We note that age and shape-based features are commonly selected features by best-performing models. Interestingly, for BraTS dataset classification, *GLCM contrast feature* is selected which highlights the contrast between cancerous and non-cancerous cells as a significant feature. However, the NSCLC dataset relies on *GLCM cluster prominence* showing the symmetry in the image as a prominent feature. The difference in different feature selection for MRI and CT scans is linked to different image fetching techniques.

**Table 10:** Model Selection for Classification: BraTS and NSCLC.

**Sr#**	**Feature Class**	**Model**	**FSM**	**FS**	**MinA**	**MaxA**	**AD**	**AUC**
**BraTS**								
**1**	GLRLM	LoR	MIr	5	66	66	0	0.769
**2**	GLRLM	MLPC	MIc	6	29	66	37	0.698
**3**	Radiomic	SVM	MIc	22	32	66	34	0.509
**4**	First Order	RFC	Fc	12	42	67	25	0.386
**5**	GLCM	DTC	Fr	13	34	62	28	0.428
**6**	Radiomic	ANN	Fr	16	31	66	35	0.425
**NSCLC**								
**1**	GLRLM	LoR	MIr	6	54	54	0	0.751
**2**	NGTDM	MLPC	MIr	all	46	54	8	0.734
**3**	NGTDM	SVM	MIc	all	32	54	22	0.531
**4**	First Order+ Shape based	RFC	MIr	7	48	58	10	0.444
**5**	NGTDM	DTC	MIr	5	38	58	20	0.382
**6**	NGTDM	ANN	MIc	2	27	52	25	0.424

**Table 11:** Model selection for regression: BraTS and NSCLC.

**Sr#**	**Feature class**	**Model**	**FSM**	**FS**	**MinA**	**MaxA**	**AD**	**AIC**
**BraTS**								
**1**	Radiomic	LR	Fc	all	52	52	0	−71.18
**2**	GLCM	MLPR	Fc	16	19	59	40	371.74
**3**	GLRLM	RFR	Fr	4	29	56	27	−10.87
**4**	First Order	DTR	MIr	15	31	58	27	34.39
**NSCLC**								
**1**	First Order	LR	Fr	10	53	53	0	−29.55
**2**	First Order	MLPR	Fc	3	50	55	5	−17.38
**3**	Shape Based	RFR	MIr	2	54	59	7	−29.14
**4**	First Order	DTR	MIr	2	54	66	12	65.26

**Table 12:** Top five features of LoR and LR model on BraTS and NSCLC dataset.

**SR#**	**Dataset**	**Model**	**FS**	**FSM**	**Top features**
**1**	BraTS	LoR	5	MIr	Age, shape-based (Maximum 2D diameter, Major Axis Length), GLRLM (Gray Level Non-Uniformity), GLCM (Contrast)
		LR	all	Fc	Age, shape-based (Least Axis Length, Major Axis Length, Minor Axis Length, Surface Volume Ratio),
**2**	NSCLC	LoR	10	MIr	First-order (Root Mean Squared, Mean, shape-based (Major Axis Length), Age, GLRLM (GrayLevelVariance)
		LR	10	Fr	Age, first-order (90th percentile), GLCM (Cluster Prominence), shape-based (Minor Axis Length, Surface Volume Ratio)

## Conclusion

The overall survival prediction of cancer patients using medical imaging is a challenging task due to the scarcity of clinical information and complex features. In this research work, we examine medical images to predict the survival time of cancer patients by using MRI/CT scanned images and age. In this regard, we explore the impact of six feature selection methods and 10 machine learning models on brain tumor and lung cancer datasets. Our analysis emphasizes using *top features* of images selected by uni-variant and mutual-information based feature selection methods. The feature selection improves the accuracy of models up to 98%. In addition, results show that GLRLM features provide the highest 66% and 54% accuracy using the logistic regression model on BraTS 2020 and NSCLC datasets, respectively. Also, we observe that random forest, decision tree, SVM, and ANN models cannot be used to train a generalized model for survival prediction. The results of these models vary with the changing distribution of training and testing data. Moreover, in-depth analysis of the best performing feature highlights the ‘age’ as the most common and contributing feature for survival prediction.

In this article, we utilize statistical methods for feature extraction. In future, we plan to test machine/deep learning-based feature extraction methods like convolutional neural network (CNN) and generative adversarial networks (GANs). Moreover, the scope of work can be extended to other diseases of breast, liver, and bone cancer.

##  Supplemental Information

10.7717/peerj-cs.1090/supp-1 Supplemental Information 1 Code and data files of the experiment

10.7717/peerj-cs.1090/supp-2 Supplemental Information 2 ReadMe file for code

10.7717/peerj-cs.1090/supp-3 Supplemental Information 3 Value of parameters used to train the model on Windows 10 using the python 3.9 installed using Anaconda 64 bit-version

## Additional Information and Declarations

### Data Availability

The following information was supplied regarding data availability: The code and data are available in the Supplemental Files.
