# Supplementary material for: Towards survival prediction of cancer patients using medical images
Source: PeerJ Comput Sci. 2022 Oct 26;8:e1090. doi: 10.7717/peerj-cs.1090 (PMC9680890; doi:10.7717/peerj-cs.1090)
Supplement: Supplemental Information 1 [file peerj-cs-08-1090-s001.zip › FinalCodeV1.2/Readme File.docx]

**Readme File**

Please follow the following steps to run the code:

1. First of all extract SPC-MI.zip file.
2. **Datasets:** We used following datasets in our research work.

- BraTS 2020 dataset are present in “brats 2020” folder
- NSCLC Dataset are present in NSCLC-Radiomics.zip

1. **Code:** To run code follow the given steps below.
   1. **BraTS 2020 dataset:** Follow the given steps to get results on BraTS 2020 dataset.

- Radiomic features are extracted using **“**RadiomicFeatures.ipynb” file.
- Hand-crafted features are extracted using “FeatureExtractor.ipynb” file.
- Benchmark results on BraTS 2020 dataset are extracted using “Results.ipynb” present in “Benchmark Brats2020” folder.
- Results using feature selection methods are extracted using “Results.ipynb” present in “Benchmark Brats2020 Feature Selection” folder.
- AUC and AIC values are also computed using CodeAUC.py and CodeAIC.py
  1. **NSCLC-Radiomics:** Follow the given steps to get results on NSCLC-Radiomics dataset.
- **Radiomic Features:** To extract radiomic features from NSCLC dataset just run “HelloORAW.py” from folder “o-raw-master”. For this you have to install Visual studio and install python module from Visual studio. Just run “python -m pip install pyrex_requirements.txt” this command and it will automatically install all libraries and then run “HelloORAW.py” file and results will be stores in “RFstore” folder.
- Benchmark results on NSCLC dataset are extracted using “Results.ipynb” present in “Benchmark NSCLC” folder.
- Results using feature selection methods are extracted using “Results.ipynb” present in “Benchmark NSCLC Feature Selection” folder.
- AUC and AIC values are also computed using CodeAUC.py and CodeAIC.py

**Note:** All excel files used to extract results are present in the respective folders. All code files are Jupyter files and self-explainable.
