# Supplementary material for: Towards survival prediction of cancer patients using medical images
Source: PeerJ Comput Sci. 2022 Oct 26;8:e1090. doi: 10.7717/peerj-cs.1090 (PMC9680890; doi:10.7717/peerj-cs.1090)
Supplement: Supplemental Information 3 [file peerj-cs-08-1090-s003.pdf]

SR#	Model	Parameters Used
1	LoR	Penalty: l2, dual=false, tolerance= 1e-4, C=1.0, fit intercep=1, intercept scaling=1
2	LRx	fit intercept=True, normalize=false, copy X=true, n jobs=none, positive=false
3	RFC	n estimators=100, criterion=gini, max depth=none, min samples split=2, min samples leaf=1, min weight fraction leaf=1, max features=sqrt, bootstrap=True, max leaf nodes=none, min impurity decrease=0, oob score=false, n jobs=none, random state=none, verbose=0, warm start=false, class weight=none, ccp alpha=0, max samples=none
4	RFR	n estimators=100, criterion=squared error, max depth=none, min samples split=2, min samples leaf=1, min weight fraction leaf=0, max leaf nodes=none, min impurity decrease=0, bootstrap=True, oob score=false, n jobs=none, random state=none, verbose=0, ccp alpha=0, max samples=none
5	DTC	criterion=gini, splitter=best, max depth=none, min samples split=2, min samples leaf=1, min weight fraction leaf=0, max features=None, max leaf nodes=none, min impurity decrease=0, ccp alpha=0, class weight= none
6	DTR	criterion=squared error, splitter=best, max depth=none, min samples split=2, min samples leaf=1, min weight fraction leaf=0, max features=None, max leaf nodes=none, min impurity decrease=0, ccp alpha=0, max features= none
7	MLPC, MLPR	hidden layer sizes=100, activation='relu', solver=adam, alpha=0.0001, batch size=auto, learning rate=constant, learning rate init=0.001, power t=0.5, max iter=200, shuffle= True, random state=none, tol=1e-4, verbose=false, warm state=false, momentum=0.9, early stopping=false, validation fraction=0.1, beta 1=0.9, beta 2=0.999, epsilom=1e-8,max fun=15000,n iter no change=10
8	SVM	C=1.0, kernel=rbf, degree=3, gamma=scale, coef0=0, shrinking=true, probability=false, tol=1e-3, cache size=200, class weight=none, verbose=false, max iter=-1, break ties=false, random state=none. decision function shape=ovr
